# Understanding the ion-induced elongation of silver nanoparticles embedded in silica

**DOI:** 10.1038/s41598-017-01145-0

**Published:** 2017-04-19

**Authors:** Ovidio Peña-Rodríguez, Alejandro Prada, José Olivares, Alicia Oliver, Luis Rodríguez-Fernández, Héctor G. Silva-Pereyra, Eduardo Bringa, José Manuel Perlado, Antonio Rivera

**Affiliations:** 1 Instituto de Fusión Nuclear, Universidad Politécnica de Madrid, José Gutiérrez Abascal 2, E-28006 Madrid, Spain; 2 Instituto de Óptica, Consejo Superior de Investigaciones Científicas (IO-CSIC), C/Serrano 121, E-28006 Madrid, Spain; 3 Centro de Microanálisis de Materiales, Universidad Autónoma de Madrid, Cantoblanco, E-28049 Madrid, Spain; 4 Instituto de Física, Universidad Nacional Autónoma de México, AP 20-364, México, D.F. 01000 Mexico; 5 IPICyT, Division de Materiales Avanzados, Camino a la presa San José 2055, San Luis Potosí, S.L.P. 78216 Mexico; 6 CONICET and Facultad de Ciencias Exactas y Naturales, Universidad Nacional de Cuyo, Mendoza, 5500 Argentina

## Abstract

In this work we have studied the elongation of silver nanoparticles irradiated with 40 MeV Bromine ions by means of *in situ* optical measurements, transmission electron microscopy and molecular dynamics simulations. The localized surface plasmon resonance of silver nanoparticles has a strong dependence on the particle shape and size, which allowed us to obtain the geometrical parameters with remarkable accuracy by means of a fit of the optical spectra. Optical results have been compared with transmission electron microscopy images and molecular dynamics simulations and the agreement is excellent in both cases. An important advantage of *in situ* measurements is that they yield an extremely detailed information of the full elongation kinetics. Final nanoparticle elongation depends on a complex competition between single-ion deformation, Ostwald ripening and dissolution. Building and validating theoretical models with the data reported in this work should be easier than with the information previously available, due to the unprecedented level of kinetic details obtained from the *in situ* measurements.

## Introduction

Metal nanoparticles (NPs) have generated a great interest in recent decades due to their remarkable optical properties, dominated by the localized surface plasmon resonances (LSPR), which are collective oscillations of the conduction electrons of the metal, excited by electromagnetic radiation^1–3^. The LSPR properties (i.e., position, intensity and linewidth) are very flexible and can be controlled by a number of parameters, such as shape, size, geometrical configuration and composition^4–6^. Hence, applications of the plasmonic NPs are equally diverse and cover a large number of areas, like photonics^7^, surface enhanced spectroscopies^8, 9^, biological imaging^10^, catalysis^11^, chemical and biological sensing^12–14^, cancer treatment^15, 16^ and energy production^17, 18^.

Nanorods are a very interesting type of plasmonic nanoparticles because, despite being a very simple shape, one can achieve a very precise control over the position and width of their LSPR simply by changing its aspect ratio^19, 20^. Geometrical asymmetry in these structures produces a splitting of the LSPR band into two modes, the longitudinal and transversal ones, which are shifted towards the red and blue (i.e., lower and larger energies), respectively^21^. There are several methods for manufacturing nanorods, among which colloidal synthesis stands as the most widely used and effective^22^. However, nanorods obtained in this way are usually contained in a solution and aligning them *a posteriori* is very difficult, though not impossible^20^. For many applications this is not a problem but in others (for example, to manufacture polarizers) it is vital that all rods are aligned.

On the other hand, ion implantation and irradiation are versatile tools for the fabrication and modification of nanostructures. Implantation and irradiation with swift heavy ions (SHIs) can be used, respectively, to synthesize^23, 24^ and elongate^25, 26^ plasmonic nanoparticles embedded in insulating matrices. This method provides little control over the size distribution and the aspect ratio, *ε*, (defined as the ratio of the large to the small axis) of the resulting rods is more limited than the one obtained with colloidal synthesis^27^. However, irradiation is a simple method that has been successfully applied to synthesize nanoparticles composed of metals and semiconductors embedded in various matrices^23, 28^. An advantageous point of the irradiation with SHIs is that nanorods fabricated in this way are perfectly aligned, because the elongation of the spherical particles occurs along the latent tracks (i.e., parallel to the irradiation direction) generated by the ions in the matrix, due to the high densities of electronic excitations induced by these ions^29, 30^. For instance, elongation of metallic nanoparticles using SHIs has been reported for cobalt NPs implanted in SiO_2_ and irradiated with iodine^31^, for gold NPs implanted in planar SiO_2_ films after irradiation with Si^32^ and Cu^33^ ions and Pt NPs formed by ion-beam synthesis in amorphous SiO_2_ irradiated with Au ions^34^. More recently, there are also several works reporting the use of swift heavy ions to elongate Ag^35, 36^, Au^37–40^, Zn^41, 42^, and Ni^43^ nanoparticles.

Unfortunately, the Physics involved in the sphere-to-rod transformation is not fully understood even if it has been extensively studied^44–46^ and some phenomenological models have been proposed^47, 48^. One of the reasons that complicate the understanding of the elongation process is the lack of accurate information during the intermediate stages of deformation. In this work, we report on the use of *in situ* transmittance measurements to study the formation of aligned silver nanorods during the irradiation with SHIs. Nearly-spherical silver NPs were obtained in a first step by implanting 2 MeV Ag ions and applying a subsequent thermal annealing. Then, the samples were irradiated with 40 MeV Br ions and the spheres were transformed into nanorods, with their larger axis aligned along the direction of the incoming ion beam. The LSPR of the silver NPs is very intense and its splitting, induced by the deformation, can be detected in the absorption spectra with high sensitivity. Hence, by fitting the spectra with a theoretical model, we were able to determine the variations of the aspect ratio as a function of the fluence, *f*.

The obtained nanoparticles were characterized by transmission electron microscopy (TEM) and optical absorption spectroscopy (OAS). The experimental optical absorption spectra were fitted with simulations performed using the transition matrix (T-matrix) method^49^, and structural information was extracted from those fits. The simulated spectra showed an excellent agreement with the experimental results. MD simulations were performed to reproduce the elongation in the initial stages of irradiation and the agreement between the simulations and structural data obtained from the fit of the optical spectra is excellent.

## Results and Discussion

**Figure 1:**
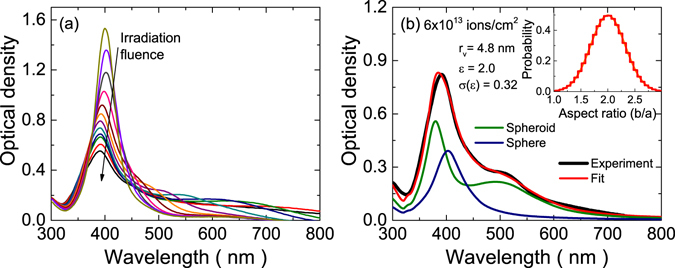
(**a**) Selected experimental optical spectra, illustrating the appearance and evolution of the second LSPR mode upon irradiation. (**b**) Typical fit of an experimental spectrum with the model described in this paper, showing the parameters obtained from the fit (*r*_*V*_, *ε*, *σ*(*ε*) and the contribution of spheroids and spheres to the total optical spectrum).

Some selected optical spectra taken during the irradiation are depicted in Fig. 1a. It can be clearly seen there that the LSPR peak is split in two as the irradiation progresses. Then, one of the new peaks red-shifts whereas the other is slightly blue-shifted. This behaviour is clearly indicative of a gradual elongation of, at least, some of the silver nanoparticles in the sample. For a more quantitative assessment, we performed the fit described in the Methods section over all the measured spectra (around 10,000 over 30 minutes of irradiation). A typical fit is illustrated in Fig. 1b; as can be seen there, the quality of the fits is very good and yields quantitative information about the geometry of the nanorods, as well as their concentration.

**Figure 2:**
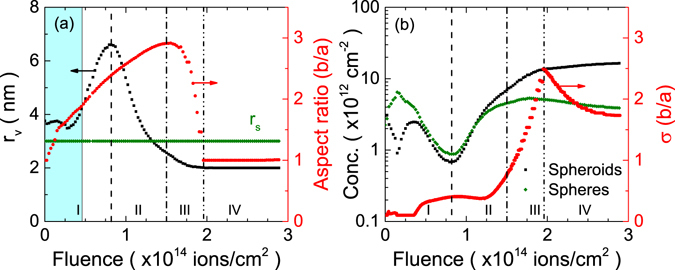
Geometrical and compositional parameters of the ensemble of nanoparticles as a function of the fluence, obtained from the fit of the optical spectra. The parameters are (**a**) the radius of the small spheres (*r*_*s*_, fixed), the volume-equivalent radius (*r*_*v*_) and average aspect ratio (*ε*) of the large rods, (**b**) the concentration of spheres and rods and the standard deviation of the distribution of aspect ratios. The cyan area represents approximately the “pure elongation” region that was used to compare with MD simulations.

The results obtained from the fits are shown in Fig. 2. There are four well-defined regions for the evolution of the nanoparticles as a function of the fluence. Region I, up to a fluence around 8 × 10^13^ cm^−2^ is characterized by a monotonical increase of the aspect ratio and the average radius of the spheroids. The latter is indicative of an Ostwald ripening produced by the irradiation. This behaviour can be understood if we consider that the energy deposited by the impinging ions is able to destroy the smaller particles and, subsequently, their constituent atoms are absorbed by the larger ones^50^. A consequence of this process is the reduction in the concentration of spheres and spheroids depicted in Fig. 2b because the larger particles “absorb” the smaller ones, growing at their expense. This region is the most interesting from a point of view of fabricating embedded nanorods and, consequently, most of the current studies have been performed exclusively in this irradiation regime^31, 47, 50–52^. However, kinetic evolution of the ensemble of nanoparticles continues for larger irradiation fluences. For example, the aspect ratio continues to increase as the irradiation progresses but the average radius of the spheroids is considerably reduced (region II, approximately up to 1.5 × 10^14^ cm^−2^). This trend indicates that the larger particles can be also dissolved upon irradiation, probably due to the cumulative damage produced by several impacts. The dissolved atoms then regrow as new particles that, depending on their size, can be also elongated. For this reason the concentration of both, spheres and spheroids, increases again as the irradiation progresses.

The reduction of the average radius continues in region III (up to 2 × 10^14^ cm^−2^) but the average aspect ratio is also considerably reduced down to one. It should be noted that this does not mean that all the rods disappear, as indicated by the increasing standard deviation of the aspect ratio (Fig. 2b) in this region. However, the number of large rods is sensibly smaller than in regions I and II. The most likely explanation for this kinetics is that even larger spheroids are also destroyed in region III, and then regrow as smaller spherical particles. Finally, the optical spectra remain nearly constant for fluences above 2 × 10^14^ cm^−2^ (region IV), meaning that the evolution of the nanoparticles reaches a plateau. This does not necessarily implies that they remain constant. It is more likely that at this point most of the remaining particles are small spherical NPs that are destroyed by the incoming ions but then regrow in a new, and similar, nanoparticle.

In summary, the final elongation of the nanoparticles (at least in this irradiation regime) is not at all a single-ion process. Instead, it is a complex competition between deformation, Ostwald ripening and dissolution. It is also worth mentioning that for all the spectra the model gives values of silver fluence very close to the actual experimental value (5 × 10^16^ cm^−2^, not shown). This means that (i) almost no atoms remain dissolved in the matrix and (ii) the model behaves very well for determining the total number of particles. So, the total number of particles is reduced in region I (supporting the Ostwald ripening) and then increases in region II (confirming the dissolution of the larger particles that regrow as smaller ones). It is worth mentioning that the designation of “spheroids” and “spheres” is only meaningful in regions I and II because beyond this point the “spheroids” have an aspect ratio close to the unity.

**Figure 3:**
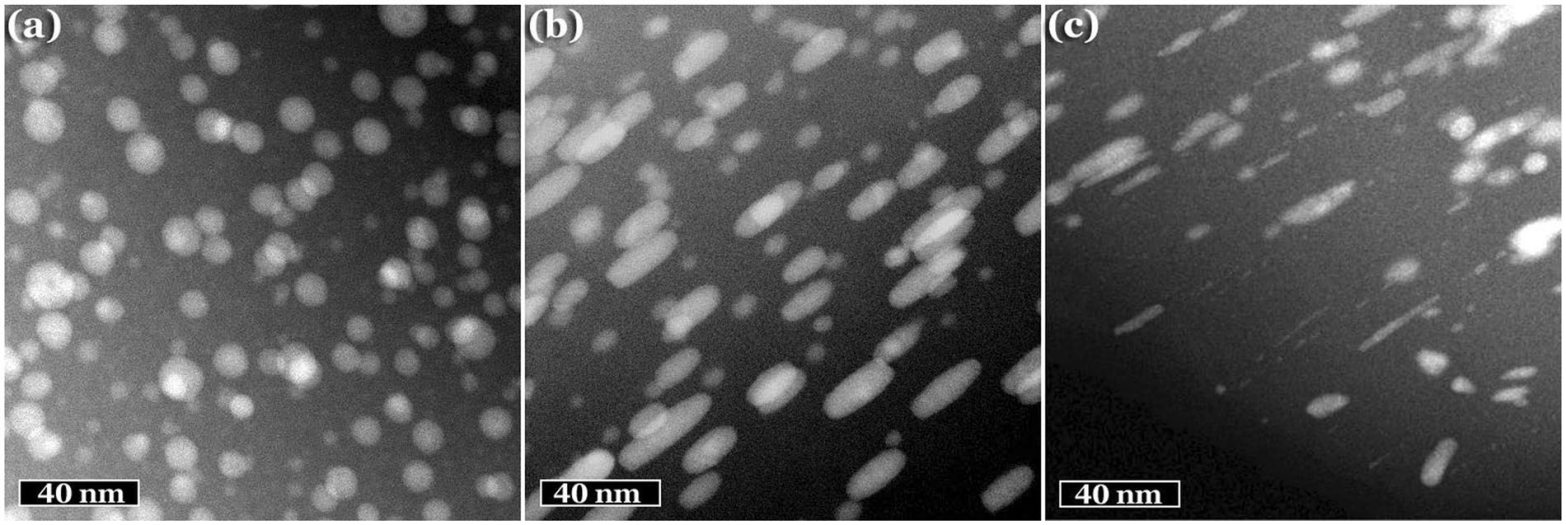
Low magnification HAADF-TEM micrographs showing the nanoparticles produced by silver implantation in silica, (**a**) as obtained and after irradiating with 40 MeV Br ions and fluences of (**b**) 8 × 10^13^ cm^−2^ and (**c**) 1.5 × 10^14^ cm^−2^. In Figures (**b,c**) the Br ion beam impinged parallel to the NP elongation.

Figure 3 shows low magnification HAADF-TEM micrographs of a cross-section of a similar sample before and after irradiation with fluences of 8 × 10^13^ cm^−2^ and 1.5 × 10^14^ cm^−2^. This provides and additional reinforcement of the results obtained from the optical spectra. It can be seen there that the sample initially contains only spherical nanoparticles (Fig. 3a) but after irradiation with a fluence of 8 × 10^13^ cm^−2^ (Fig. 3b) the elongation is clearly appreciable. However, if the sample is irradiated with a larger fluence some nanoparticles are destroyed or heavily modified (Fig. 3c). The TEM images also show (Fig. 3b) that the smaller diameter of the elongated nanoparticles is very similar in all cases, irrespectively of the total size of the particle. This effect is not just present in our samples, it is a consequence of the irradiation with SHIs and is related to the radius of the latent tracks^48^. In other words, the minor axis of the nanorods cannot be lower than the radius of the latent tracks produced by the ions in the matrix, which is around 3.8 nm in our samples^53^. This implies that NPs smaller than this size cannot be elongated and justifies the inclusion of small spherical particles in our model.

**Figure 4:**
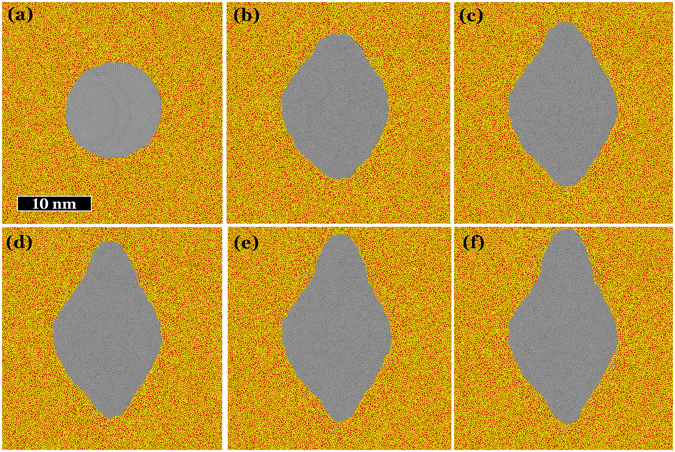
Evolution of silver nanoparticles embedded in silica 100 ps after energy deposition in a hot cylinder of radius *a* = 3.5 nm oriented with its axis in the vertical direction. Simulations were performed for an electronic stopping power of 8 keV/nm. Each image shows a thin central slide of the box used for MD simulations, (**a**) prior to irradiation and after (**b**) 2, (**c**) 4, (**d**) 6, (**e**) 8 and (**f**) 10 ion impacts.

In order to better understand the elongation process we used MD simulations to study the evolution of silver NPs (*R* = 5.9 nm) embedded in silica, for an increasing number of ion impacts. The obtained results are depicted in Fig. 4, it is evident there that each ion induces just a small elongation and only the cumulative effect of various ions can produce nanorods with a large aspect ratio. Ions with a larger stopping power are expected to produce larger elongations with only one impact but even in this case, several ions are required to generate rods with a considerable aspect ratio^45^. The MD simulations allow us to understand the physical mechanisms behind the NP elongation. Upon energy deposition a shock wave is generated in the silver NP. When the wave reaches the NP-silica interface only a fraction of the energy transported can be dissipated into the silica; thus, most of the energy is reflected back to the centre of the NP. This process lasts a few cycles until the wave is damped. Then, the silver NP reaches a high temperature as well as the silica region within the track (for *S*_*e*_ = 6 keV/nm, at 11 ps, the NP temperature reaches 9000 K, whereas the silica temperature in the track region is around 2500 K). In these conditions, the NP-silver interface experiences a huge tensile stress (around 3 GPa for *S*_*e*_ = 6 keV/nm). This stress is the driving force behind silver atom movement. Effective silver transport only occurs in the hot silica region (i.e., along the ion trajectory). This is possible due to the fact that upon the energy deposition in the hot cylinder a collective atom motion outwards the track region takes place. The liberated space at the interface is rapidly occupied by the highly mobile silver atoms. This process is repeated after every ion impact. The result of every ion impact is a small elongation along the ion trajectory. The cumulative effect of several ions results in a significant elongation, as shown in the figure.

**Figure 5:**
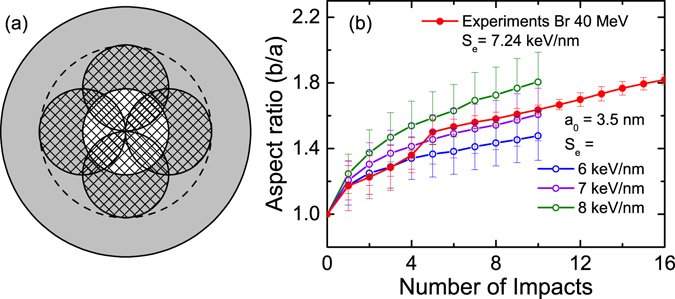
(**a**) Schematic representation of the NP region where the ion impacts produce a meaningful elongation. (**b**) Evolution of the aspect ratio as a function of the number of impacts. Solid symbols represent the experimental results and open symbols the MD simulations.

The comparison of experimental results and MD simulations cannot be done directly. Fitting of the optical spectra with the model and MD simulations yield the geometrical parameters of the nanorods as a function of fluence and the number of impacts, respectively. To obtain the number of impacts affecting a nanoparticle for a given fluence the latter must be multiplied by an area. One might be tempted to use the NP’s transversal area but this choice will give wrong results because an ion that just “touches” the NP border will unlikely produce an effective elongation. Hence, to do the conversion we have assumed that the ion track (3.8 nm in this case^53^) must, at least, overlap with the nanoparticle long axis. This implies that the effective area to produce elongation is equal to the track area (white region in Fig. 5a). One additional point to consider is that MD simulations of a single particle cannot represent the Ostwald ripening. Hence, we have restricted the comparison to the region of “pure elongation”; i.e., where the only meaningful effect is the elongation (roughly the cyan region in Fig. 2a).

The resulting experimental aspect ratio as a function of the number of impacts is shown in Fig. 5b (solid symbols) for the lower fluences. This is compared with MD simulations performed for three different values of the electronic stopping power and up to ten ion impacts (because experimental results indicate that Ostwald ripening becomes important beyond this point). We recall that the experimental value of Se (at the sample surface) is 7.24 keV/nm. Except for the first few impacts, the agreement between the experiments and the curve corresponding to the theoretical *S*_*e*_ = 7 keV/nm, which is remarkable considering all the approximations that have been made. The simulations that agrees better for the first few impacts correspond to the curve obtained for *S*_*e*_ = 6 keV/nm but it should be noted that the fitting is very complicated for low aspect ratios because the two LSPR peaks overlap. Hence, we are convinced that this result is probably more related to a limitation of the fitting than to a real physical effect.

The good agreement between the optical measurements, the TEM images and the MD simulations indicate that *in situ* optical techniques provide accurate results on the geometry and concentration of the nanoparticles as a function of the irradiation fluence. One limitation of this technique is that, since it is based on the fitting of the LSPR, it is only effective for plasmonic materials (mainly silver and gold). However, beyond this restriction it is hard to overestimate the level of kinetic details offered by this technique. The final elongation produced by swift heavy ions is a complex competition between the single-ion elongation, Ostwald ripening and dissolution. Thanks to the measurements carried out in this work we have been able to identify four different regions whereas all works that we are aware of have focused only in the first region.

## Methods

### Specimen preparation and characterization

High-purity silica glass plates (20 × 20 × 1 mm^3^) with a content of hydroxyl radicals (OH) lower than one part per million (ppm) and total impurity content less than 20 ppm (with no individual impurity content greater than 1 ppm), were implanted at room temperature with 2 MeV Ag ions at a fluence of 2 × 10^16^ cm^−2^, using the 3 MV Tandem accelerator (NEC 9SDH2 Pelletron) of the Instituto de Física, Universidad Nacional Autónoma de México (UNAM). Afterwards, the samples were annealed in air during an hour, at a temperature of 600 °C, in order to induce the nucleation of silver nanospheres.

**Figure 6:**
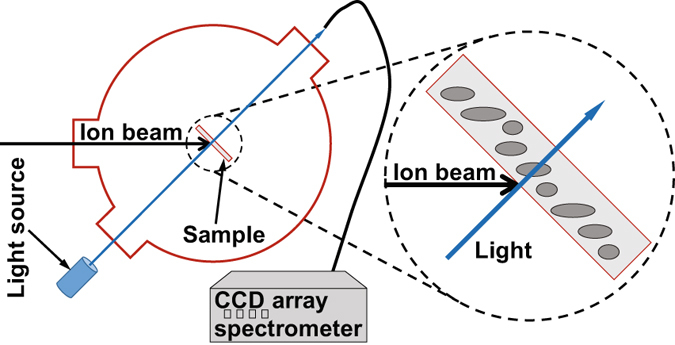
Schematic representation of the setup used for *in situ* transmittance measurements. Samples can be irradiated with swift heavy ions and simultaneously illuminated with white light (blue arrows). Transmitted light is collected by an optical fiber and guided to a CCD array spectrometer.

Spherical NPs were then elongated by irradiating the samples with a 40 MeV Br ion beam up to a fluence of 3 × 10^14^ cm^−2^, in a standard scattering chamber connected to a 5 MV tandem accelerator in the Centro de Microanálisis de Materiales (CMAM)^54^. The beam homogeneity was carefully checked to be within 10% by means of the ionoluminescence induced in a silica sample monitored with a 12-bit CCD camera. The samples were tilted to 45°, in order to put their surface perpendicular to the incident light (see Fig. 6). Currents in the range 10–30 nA were used, to avoid overheating of the samples.

A schematic representation of the setup used for the *in situ* measurements is depicted in Fig. 6. The absorption spectra were measured by sending the light produced by a halogen lamp through a silica window port placed at 45° with respect to the ion beam and collected through a similar port after passing through the sample. The collected light was focused with a 25-mm-diameter, 4-cm-focal-length silica lens onto a silica optical fiber of 1 mm diameter. The light was then guided to a compact spectrometer, QE6500 (Ocean Optics Inc.), configured with a multichannel array detector for measuring simultaneously the whole spectrum in the range 200–850 nm with a spectral resolution better than 2 nm. The light integration time in all the measurements was 1 s. Finally, transmission electron microscopy, in a high-angle angular dark field (HAADF-TEM) configuration, was performed using a JEOL 2010F-UHR microscope operating at 200 kV (1.9 Å point-to-point resolution) and equipped with a GATAN digital micrograph system for image acquisition.

### Spectra fitting

For the simulations we represented the nanorods by means of spheroids because this shape provides an excellent approximation of the NPs elongated with SHIs^26, 27^. Then, the optical spectra were fitted with T-matrix simulations, performed with Mishchenko’s T-matrix codes^49^ and a procedure that we have described elsewhere^26, 55^. The bulk dielectric function values reported by Johnson and Christy^56^ were used for the calculations. It was assumed, based on our previous experience^26, 27^, that the samples contained a mixture of small spheres and larger spheroids. Within the range of size in our samples (<15 nm), the effect of *ε* on the optical response is far more important than that of the size; hence, we assumed single values for the radius of the sphere and for the volume-equivalent radius of the spheroid, *r*_*v*_, (i.e., the radius of an sphere having the same volume). Moreover, after some preliminary fits, we used a fixed radius of 3 nm for the spheres. Finally, we used a Gaussian distribution for the aspect ratio of the spheroids. In summary, the fit allowed us to obtain *r*_*v*_, the mean and the standard deviation of *ε*, as well as the concentration of the spheres and spheroids. The optimization was performed by means of the L-BFGS-B algorithm, a limited memory quasi-Newton algorithm for solving large nonlinear optimization problems with simple bounds on the variables^57, 58^.

### Molecular dynamics simulations

Molecular dynamics (MD) simulations were carried out with the parallel computing code MDCASK. We used the Feuston-Garofalini potential^59^ to reproduce Si-Si, O-O and Si-O interactions in silica, the embedded-atom method (EAM) potential^60^ for silver interactions and the Born-Mayer-Huggins (BMH) potential^61^ for Si-Ag and Ag-O interactions. These potentials were modified with the Ziegler, Biersack and Littmark (ZBL) potential to represent the short range interactions (strong force)^62^. The simulation boxes contained more than 1.7 × 10^6^ atoms (30 × 30 × 28.6 nm^3^). The initial box was made from a cristobalite structure^63^ that was heated to 5000 K during 50 ps. Then, it was cooled down to 300 K by a repetitive process consisting of reducing the temperature in steps of 1000 K and keeping each temperature constant during 25 ps. Once cooled down, we removed from the box all the atoms contained within a sphere with a radius of 5.1 nm and centered in the origin and introduced in this position a silver sphere with a radius of 5 nm and a lattice parameter of 3.33 Å^64^. The temperature of the silver cluster was increased linearly up to 600 K during 25 ps, maintained constant during 25 ps and reduced linearly down to 300 K during 25 ps. Finally, the box temperature (for both, silica and silver) was maintained at 300 K during 25 ps. The silver nanoparticle was expanded during those processes, yielding a stable sphere with a radius of 5.9 nm.

The swift heavy ion irradiation was reproduced by a procedure extensively used in the literature for several materials^65–71^. It is based on the assumption that the energy deposited from the incoming ion in the electron system is eventually transmitted to the lattice atoms very rapidly. Thus, we set the initial kinetic energy per unit length of the atoms in a cylindrical region around the ion trajectory (hot cylinder) equal to the electronic stopping power of the ion (in this paper 6, 7 and 8 keV/nm). The radius of the hot cylinder is variable and it is related to the energy density induced by the ion around its trajectory and, consequently, to the ion velocity and the electron-phonon coupling factor. For the ions used in this work we have found that the optimal radius is *a* = 3.5 nm. The cylinder axis coincides with the irradiation direction. A thermal bath at 300 K is maintained by rescaling the velocity of the atoms located in the lateral sides beyond an imaginary cylinder of radius 14.28 nm centered and parallel to the hot cylinder. Simulations are carried out during 75 ps with a time step of 0.5 fs making use of periodic boundary conditions. At this time, we obtain a cold silica box with a hot embedded silver nanoparticle. This is due to poor heat transmission at the silver-silica interface. No significant evolution of the silver NP (regarding aspect ratio determination) takes place. In order to speed up the cooling process, for times beyond 75 ps we include a Langevin frictional force^72–74^ to the silver atoms with a target temperature of 300 K and a damp factor chosen in four different time intervals as: (i) 75 ps from 75 to 93.75 ps, (ii) 37.5 ps from 93.75 to 112.5 ps, (iii) 18.75 ps from 112.5 to 131.25 ps and (iv) 0.5 fs from 131.25 to 150 ps. The final result is a cold and relaxed box containing a deformed (i.e., elongated) silver nanoparticle. The effect of multiple ion impacts was simulated by repeating several times the irradiation and relaxation process with the box obtained from the previous simulation.

## Conclusions

In this work we have shown that optical techniques are an excellent tool for studying *in situ* the elongation of plasmonic nanoparticles irradiated with SHIs, due to the strong dependence of the LSPR on the particle shape and size. The accuracy of the geometrical information obtained from the fit of the optical spectra is remarkable, particularly for aspect ratios larger than 1.5 because then the two LSPR peaks can be clearly resolved. Moreover, these measurements can give an extremely detailed information of the full elongation kinetics. Based on the results obtained from samples containing silver NPs and irradiated with 40 MeV Br ions, we have concluded that the final NP elongation depends on a complex competition between single-ion deformation, Ostwald ripening and dissolution. Optical results have been compared with TEM images and MD simulations and in both cases the agreement is excellent. The level of kinetic details obtained from the *in situ* measurements is unprecedented. Hence, we hope that with the data reported in this work it will be easier to develop comprehensive theoretical models able to represent the full kinetics of evolution for NPs irradiated with SHIs.
